# Supplementary material for: Cell type-specific proximity labeling of organ secretomes reveals energy balance-dependent proteomic remodeling
Source: bioRxiv. 2026 Jan 12:2026.01.11.698831. Preprint. [Version 1] doi: 10.64898/2026.01.11.698831 (PMC12871138; doi:10.64898/2026.01.11.698831)
Supplement: Supplement 1 [file NIHPP2026.01.11.698831v1-supplement-1.pdf]

bioRxiv preprint doi: https://doi.org/10.64898/2026.01.11.698831; this version posted January 12, 2026. The copyright holder for this preprint (which was not certified by peer review) is the author/funder, who has granted bioRxiv a license to display the preprint in perpetuity. It is made available under aCC-BY 4.0 International license.

Supplementary information

Cell type-specific proximity labeling of organ secretomes reveals energy balance-dependent proteomic remodeling.

Kaja Plucińska,[1,2] Charlotte R. Wayne,[1,3] Henry Sanford,[1,3] Boby Mathew,[1,4] Nathalie Ropek,[3] Stephanie M. Adaniya,[3,5] Corey Model,[2,6] Nicolás Gómez-Banoy,[2,7,8] Ksenia Morozova,[2] Xiongwen Cao,[4,9] Jeffrey M. Friedman,[10] Ken H. Loh,[4,10,*] Paul Cohen,[2,*] Ekaterina V. Vinogradova[3,*]

[1]These authors contributed equally.

[2] Laboratory of Molecular Metabolism, The Rockefeller University, 1230 York Avenue, New York, NY 10065, USA

[3] Laboratory of Chemical Immunology and Proteomics, The Rockefeller University, 1230 York Avenue, New York, NY 10065, USA

[4] Department of Comparative Medicine, Yale University, School of Medicine, New Haven, CT 06520, USA

[5] Current position: Department of Biochemistry, University of Washington, Seattle, WA 98195, USA

[6] Current position: Department of Chemistry, Stanford University, Stanford, CA 94305, USA

[7] Division of Endocrinology, Department of Medicine, Memorial Sloan Kettering Cancer Center, New York, New York 10065, USA

[8] Division of Endocrinology, Diabetes and Metabolism, Department of Medicine, Weill Cornell Medicine, New York, New York 10065, USA

[9] Current position: Shanghai Key Laboratory of Regulatory Biology, Institute of Biomedical Sciences, School of Life Sciences, East China Normal University, Shanghai 200241, China

[10] Laboratory of Molecular Genetics, The Rockefeller University, 1230 York Avenue, New York, NY 10065, USA

[*] Co-corresponding authors

Contacts:

huaijinkenleon.loh@yale.edu

pcohen@rockefeller.edu

vinograd@rockefeller.edu

bioRxiv preprint doi: https://doi.org/10.64898/2026.01.11.698831; this version posted January 12, 2026. The copyright holder for this preprint (which was not certified by peer review) is the author/funder, who has granted bioRxiv a license to display the preprint in perpetuity. It is made available under aCC-BY 4.0 International license.

(A) Supplementary figures.

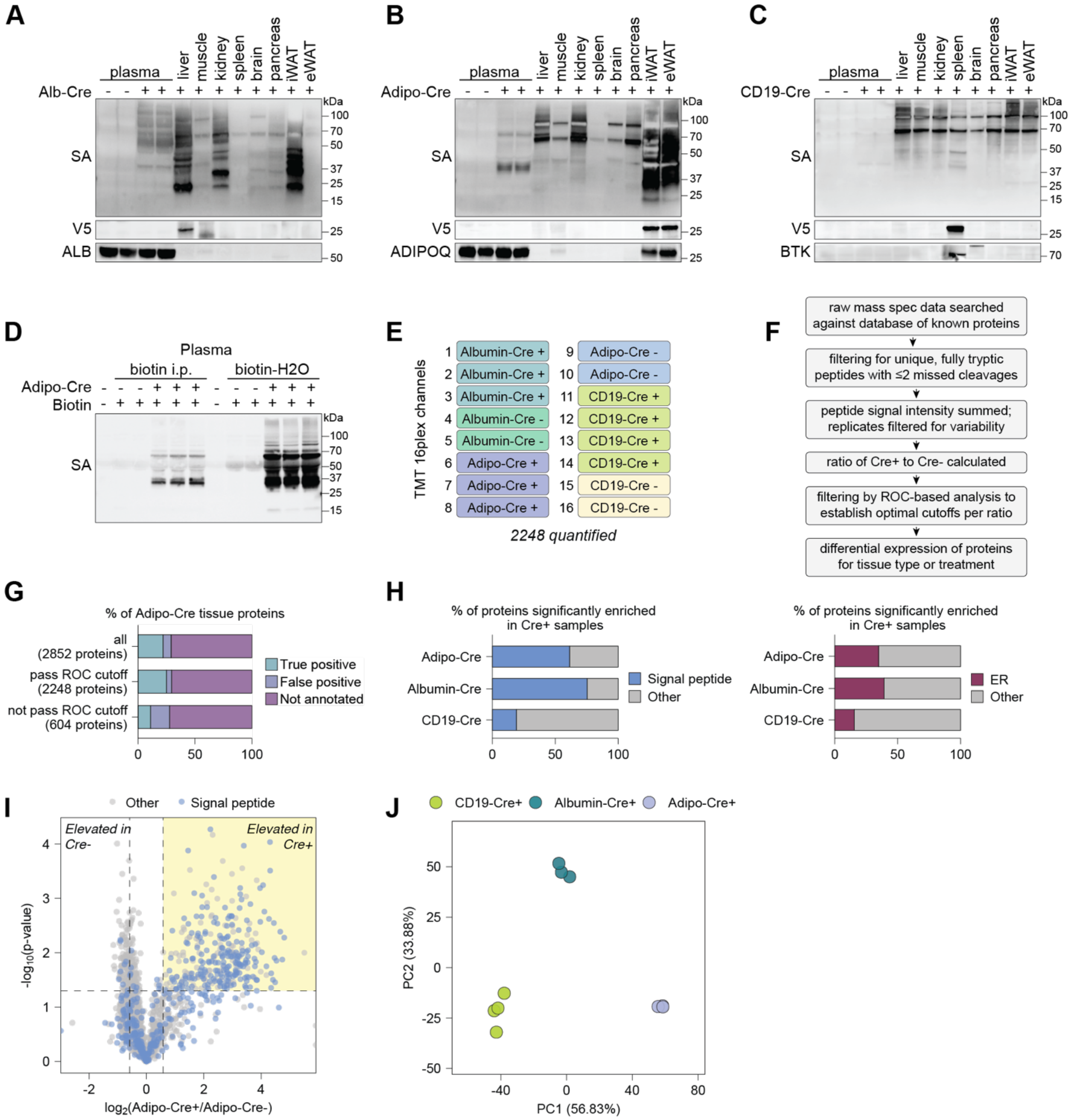

Figure S1. Characterization of a genetically encoded TurboID-KDEL in three tissue types, related to Figure 1.

(**A-C**) Protein biotinylation and V5 expression in various tissue types and plasma assessed by Western blot analysis in (A) Albumin-TurboID^{KDEL}, (B) Adiponectin-TurboID^{KDEL}, and (C) CD19-TurboID^{KDEL} mice. (**D**) Comparison of protein biotinylation efficiency in plasma from Adipo-TurboID^{KDEL} mice treated with biotin (1 mg/mL) either intraperitoneally (i.p.) or in drinking water over the course of 7 days.

bioRxiv preprint doi: https://doi.org/10.64898/2026.01.11.698831; this version posted January 12, 2026. The copyright holder for this preprint (which was not certified by peer review) is the author/funder, who has granted bioRxiv a license to display the preprint in perpetuity. It is made available under aCC-BY 4.0 International license.

(**E**) Representative TMT channel assignments for a 16-plex LC-MS/MS/MS experiment comparing three tissue types under basal conditions. The number of proteins passing the ROC cutoff is also shown.

(**F**) General filters used for analysis of tissue mass spectrometry data.

(**G**) Representative bar plot showing annotation of proteins passing and failing ROC-based filtering in Adipo-Cre samples. See supplementary methods for more details.

(**H**) Bar plot showing percentage of significantly enriched proteins (p-value < 0.05, FC > 1.5; corresponding to the highlighted region in I) annotated as secreted by SignalP and Outcyte (left) or ER by Gene Ontology annotation (right).

(**I**) Representative volcano plot showing $\log_2$ fold changes of protein expression between Adipo-Cre+ and Adipo-Cre- samples. Dashed lines represent cutoffs of p-value < 0.05 and fold-change (FC) > 1.5. Signal peptides are labeled based on OutCyte predictions.

(**J**) Principal component analysis of proteomic data from Cre+ samples. Protein signal intensity values were $\log_2$ transformed before analysis.

bioRxiv preprint doi: https://doi.org/10.64898/2026.01.11.698831; this version posted January 12, 2026. The copyright holder for this preprint (which was not certified by peer review) is the author/funder, who has granted bioRxiv a license to display the preprint in perpetuity. It is made available under aCC-BY 4.0 International license.

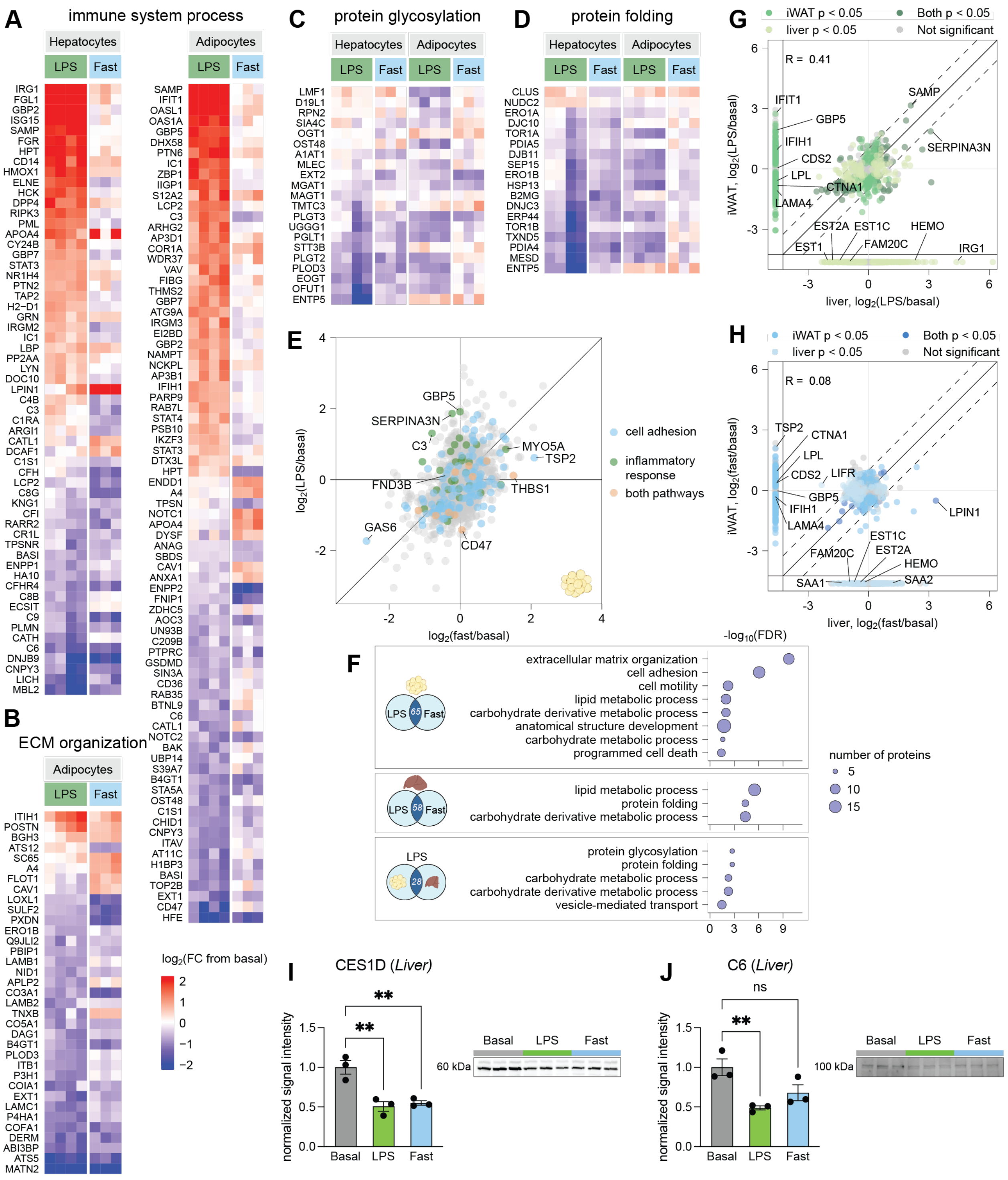

Figure S2. Comparison of proteomic responses to negative energy balance in hepatocytes and adipocytes, related to Figure 2.

(**A-D**) Heat maps showing differentially expressed proteins contributing to (A) immune system process, (B) extracellular matrix (ECM) organization, (C) protein glycosylation, and (D) protein folding GO-term

bioRxiv preprint doi: https://doi.org/10.64898/2026.01.11.698831; this version posted January 12, 2026. The copyright holder for this preprint (which was not certified by peer review) is the author/funder, who has granted bioRxiv a license to display the preprint in perpetuity. It is made available under aCC-BY 4.0 International license.

enrichment in adipocytes or hepatocytes. Data is presented as $\log_2$(fold-change) compared to basal condition.

(**E**) Scatterplot showing correlation of $\log_2$(fold-change) values for protein expression changes in adipocytes from LPS-treated or fasted Adipo-TurboID^{KDEL} mice relative to basal conditions. Proteins belonging to selected pathways are colored.

(**F**) Gene ontology (GO) analysis of pathways enriched at the intersection of proteins significantly downregulated in multiple conditions, including 66 proteins downregulated in adipocytes following LPS and fasting, 58 downregulated in hepatocytes in LPS and fasting, and 28 downregulated in both adipocytes and hepatocytes following LPS.

(**G-H**) Scatterplots of $\log_2$(fold-change) values for protein expression changes in liver (x) or iWAT (y) following LPS treatment (G) or fasting (H) compared to basal condition.

(**I-J**) Validation by Western blot of CES1D (I) and C6 (J) downregulation in response to LPS and fasting in liver. Western blot band intensity was normalized to the total intensity of the corresponding lane in a stain-free gel image. Comparison performed by one-way ANOVA with Dunnett's multiple comparisons test (ns, $p > 0.05$; **, $p < 0.01$).

bioRxiv preprint doi: https://doi.org/10.64898/2026.01.11.698831; this version posted January 12, 2026. The copyright holder for this preprint (which was not certified by peer review) is the author/funder, who has granted bioRxiv a license to display the preprint in perpetuity. It is made available under aCC-BY 4.0 International license.

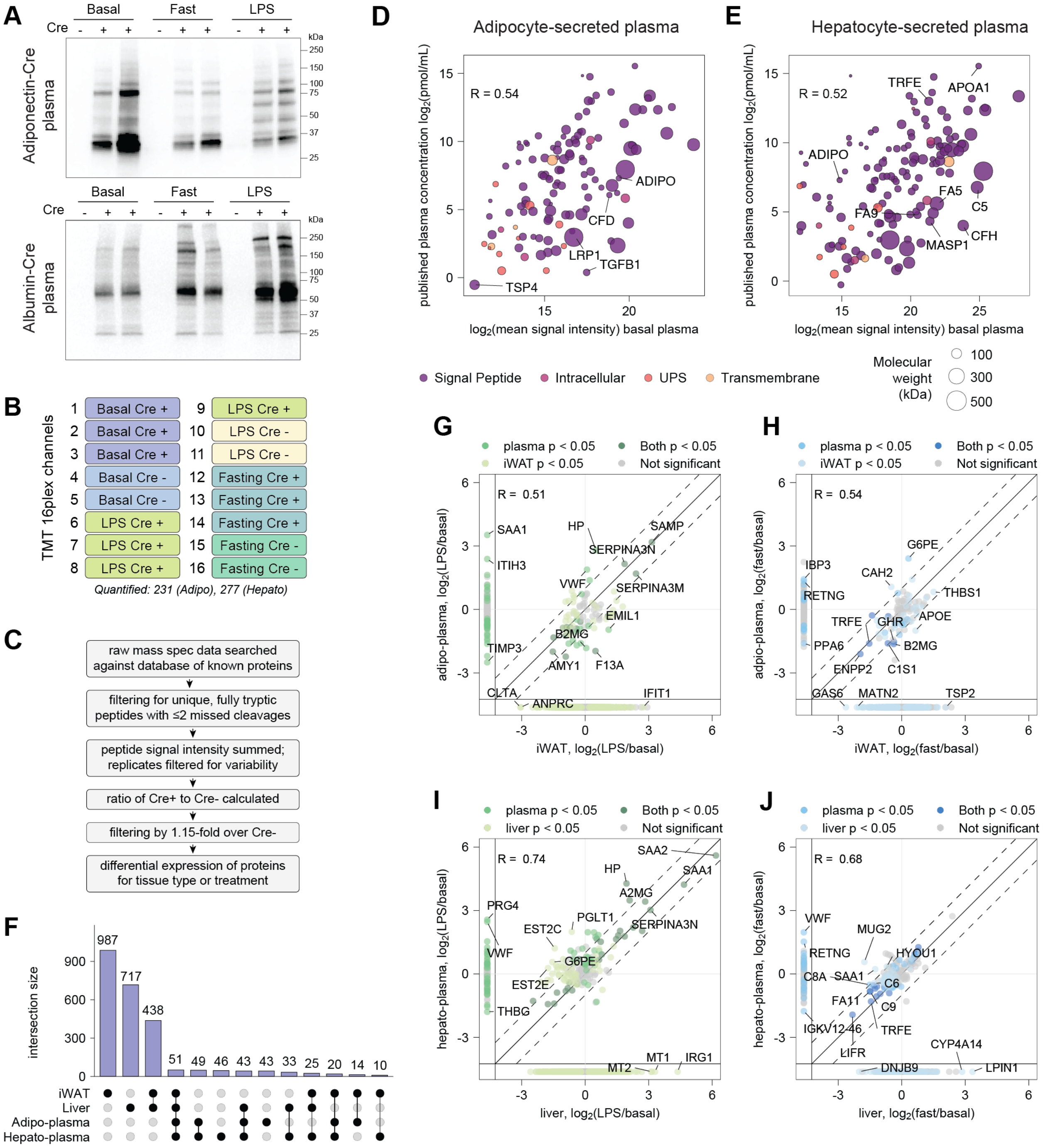

Figure S3. Hepato-plasma and adipo-plasma proteome responses to fasting or inflammation-induced anorexia, related to Figure 3.

(**A**) Western blot showing TurboID-catalyzed biotinylation of proteins identified in plasma from biotin-treated Adipo- and Albumin-TurboID^{KDEL} mice under basal and negative energy balance (fasting, LPS) conditions.

bioRxiv preprint doi: https://doi.org/10.64898/2026.01.11.698831; this version posted January 12, 2026. The copyright holder for this preprint (which was not certified by peer review) is the author/funder, who has granted bioRxiv a license to display the preprint in perpetuity. It is made available under aCC-BY 4.0 International license.

(**B**) Representative TMT channel assignment for a 16-plex LC-MS/MS/MS experiment comparing plasma proteomes from three conditions (basal, LPS, and fasting) in mice with and without a cell-type-specific Cre driver. The number of proteins passing the enrichment cutoff is also shown.

(**C**) Filters used for analysis of plasma mass spectrometry data.

(**D-E**) Scatterplots of $\log_2$-transformed mean signal intensity values (x) for proteins quantified in basal adipo-plasma (D) and hepato-plasma (E) versus mean published absolute plasma concentrations (y). Size of points represents molecular weight in kilodaltons. Colors show SignalP and Outcyte secretion predictions.

(**F**) Upset plot of overlap in protein quantifications by TurboID-TMT between adipocyte and hepatocyte tissues and plasma. Intersections with fewer than 10 proteins are not shown.

(**G-J**) Scatterplots of $\log_2$(fold-change) values for expression changes in adipocyte (F-G) or hepatocyte (H-I) secreted proteins following LPS treatment (left) or fasting (right) compared to basal condition in plasma (x) and tissue (y) samples. Proteins quantified in one sample (tissue or plasma) are shown on margins. R represents Pearson's correlation coefficient.

bioRxiv preprint doi: https://doi.org/10.64898/2026.01.11.698831; this version posted January 12, 2026. The copyright holder for this preprint (which was not certified by peer review) is the author/funder, who has granted bioRxiv a license to display the preprint in perpetuity. It is made available under aCC-BY 4.0 International license.

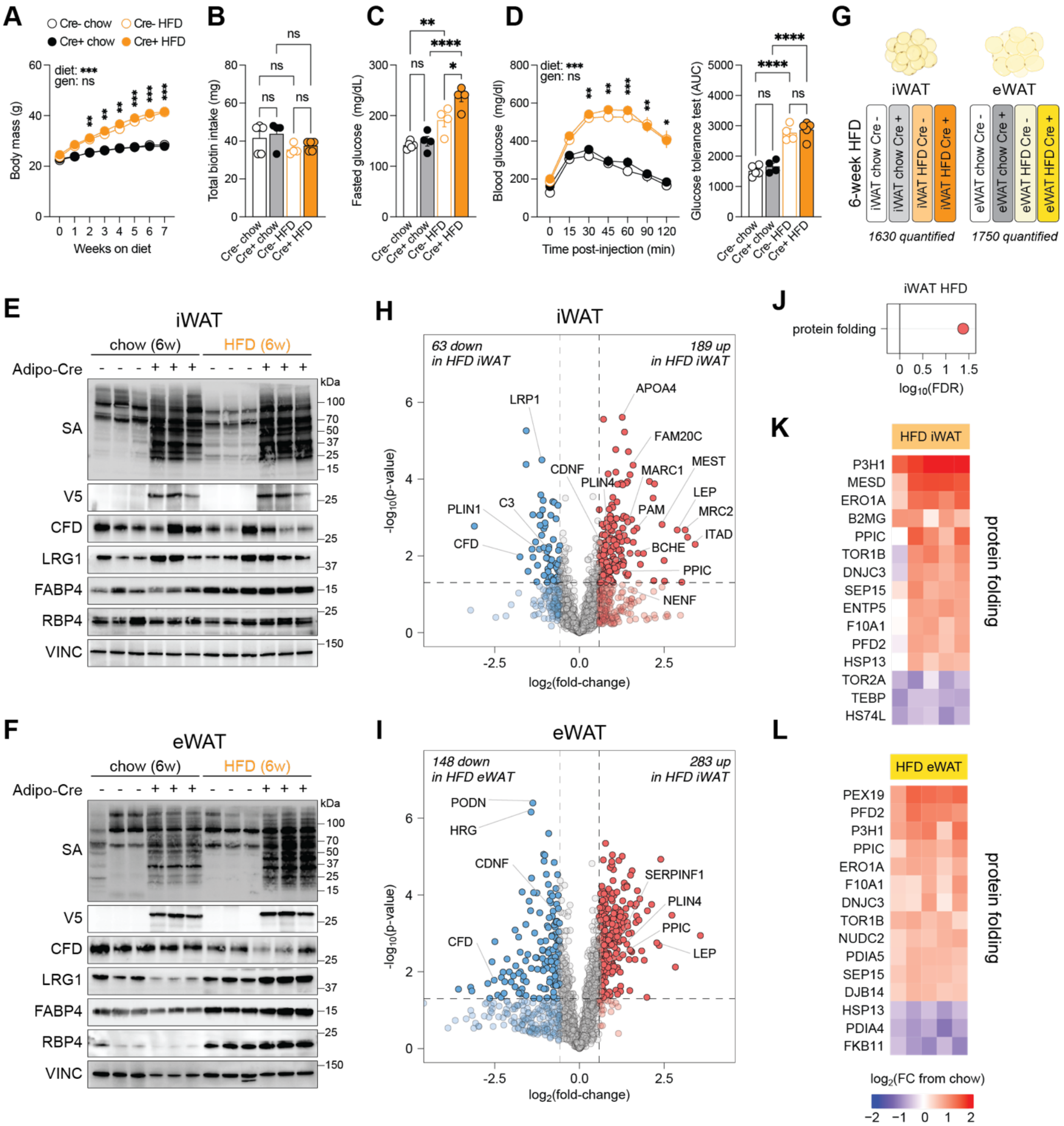

Figure S4. Proteomic responses of inguinal and epidydimal adipocytes in Adipo-TurboID^{KDEL} mice to early obesity, related to Figure 4.

(**A**) Body weights of Adipo-TurboID^{KDEL} mice fed HFD or chow for 6 weeks. Biotin (1.5 mg/mL) was administrated in drinking water for 7 days on the last week of feeding. Conditions were compared to basal by two-way ANOVA with Tukey's multiple comparisons test (ns, $p > 0.05$, not shown; *, $p < 0.05$; **, $p < 0.01$). Error bars show mean ± SEM; n = 4-6 mice per condition.

bioRxiv preprint doi: https://doi.org/10.64898/2026.01.11.698831; this version posted January 12, 2026. The copyright holder for this preprint (which was not certified by peer review) is the author/funder, who has granted bioRxiv a license to display the preprint in perpetuity. It is made available under aCC-BY 4.0 International license.

(**B**) Total biotin intake in mice after 7 days. Conditions were compared to basal ordinary one-way ANOVA with Šidák multiple comparisons test (ns, $p > 0.05$). Error bars show mean ± SEM; n = 4-7 mice per condition.

(**C**) Fasted glucose in mice fed chow or HFD for 6 weeks. Conditions were compared to basal ordinary one-way ANOVA with Šidák multiple comparisons test (ns, $p > 0.05$; *, $p < 0.05$; **, $p < 0.01$; ****, $p < 0.0001$). Error bars show mean ± SEM; n = 4-7 mice per condition.

(**D**) Glucose tolerance in mice fed chow or HFD for 6 weeks and area under curve (AUC). Conditions were compared to basal by two-way ANOVA with Tukey's multiple comparisons test (ns, $p > 0.05$, not shown; *, $p < 0.05$; **, $p < 0.01$; ***, $p < 0.001$). Error bars show mean ± SEM; n = 4-7 mice per condition. AUC was compared using ordinary one-way ANOVA with Šidák multiple comparisons test (ns, $p > 0.05$; ****, $p < 0.0001$). Error bars show mean ± SEM; n = 4-7 mice per condition.

(**E-F**) Western blot validation of protein biotinylation using streptavidin-HRP, tissue-specific V5 expression, and levels of known adipokines including CFD, LRG1, FABP4, and RBP4 in bulk iWAT (E) and eWAT (F) from Adipo-TurboID^{KDEL} mice after 6 weeks of HFD feeding. Vinculin (VINC) was used as a loading control.

(**G**) Representative TMT channel assignment for a 16-plex LC-MS/MS/MS experiment comparing proteomic changes induced by 6-week HFD vs. chow in iWAT or eWAT adipocytes from Adipo-TurboID^{KDEL} Cre+ and Cre- mice (technical replicates not shown). The number of proteins passing the ROC cutoff in each experiment is also shown.

(H-I) Volcano plots showing $\log_2$ fold changes in secretory pathway protein expression in iWAT (H) and eWAT (I) samples following HFD feeding for 6 weeks relative to basal. Dashed lines represent cutoffs of p-value < 0.05 and fold-change > 1.5.

(**J**) Biological process over-represented in gene set enrichment affected by HFD feeding in iWAT-derived adipocytes at 6 weeks of HFD.

(**K-L**) Heat maps showing differentially expressed proteins contributing to protein folding GO-term enrichment in iWAT (K) and eWAT (L) samples following HFD feeding for 6 weeks. Data is presented as $\log_2$(fold-change) compared to the chow condition.

bioRxiv preprint doi: https://doi.org/10.64898/2026.01.11.698831; this version posted January 12, 2026. The copyright holder for this preprint (which was not certified by peer review) is the author/funder, who has granted bioRxiv a license to display the preprint in perpetuity. It is made available under aCC-BY 4.0 International license.

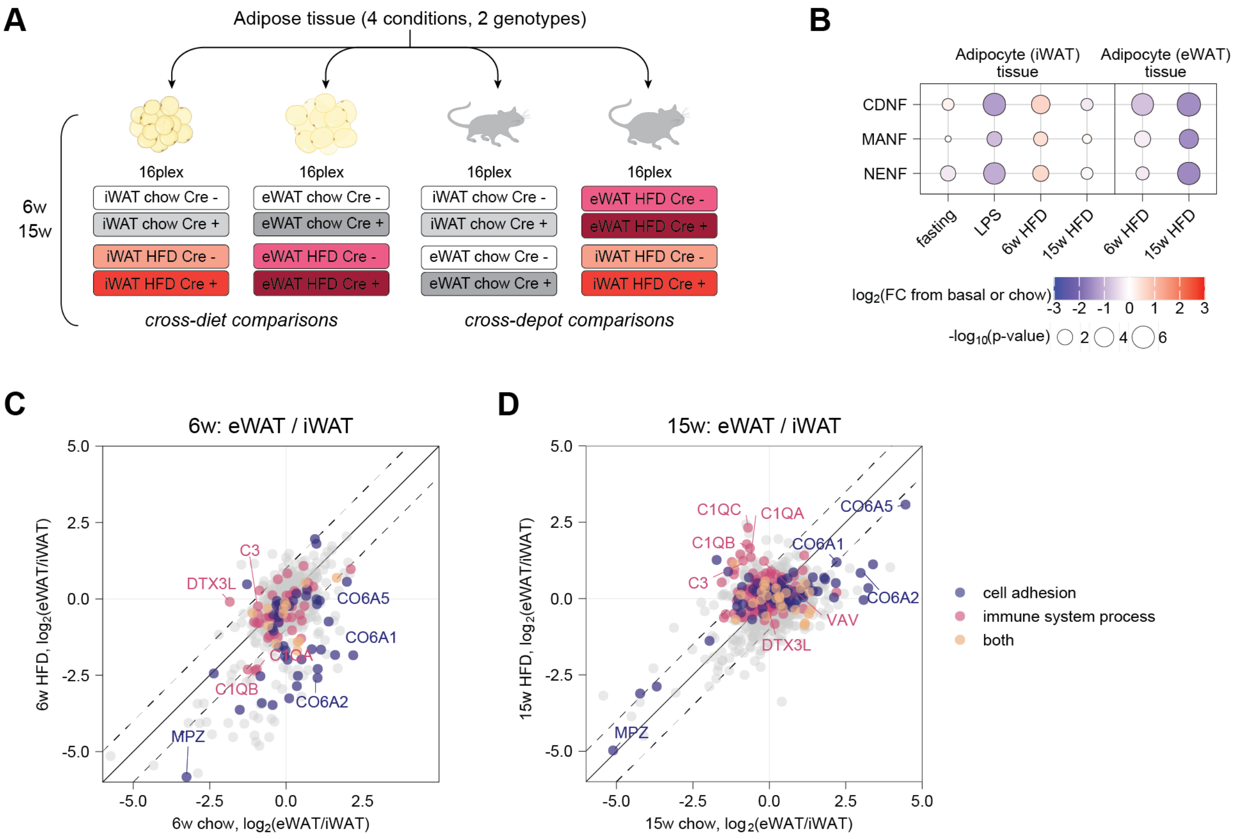

Figure S5. Depot-specific protein remodeling in inguinal and epididymal adipocytes in early and advanced obesity, related to Figure 5.

(**A**) Schematic showing TurboID-TMT MS experiments for cross-diet and cross-depot comparisons (technical replicates not shown).

(**B**) Normalized mean signal intensity values for CDNF, MANF, and NENF proteins across high-fat diet experiments and depot.

(**C-D**) Correlational analysis of $\log_2$ fold changes in protein expression following chow and HFD feeding for 6 weeks (C) and 15 weeks (D) in inguinal compared to epididymal adipocytes. Proteins belonging to selected pathways are colored.

bioRxiv preprint doi: https://doi.org/10.64898/2026.01.11.698831; this version posted January 12, 2026. The copyright holder for this preprint (which was not certified by peer review) is the author/funder, who has granted bioRxiv a license to display the preprint in perpetuity. It is made available under aCC-BY 4.0 International license.

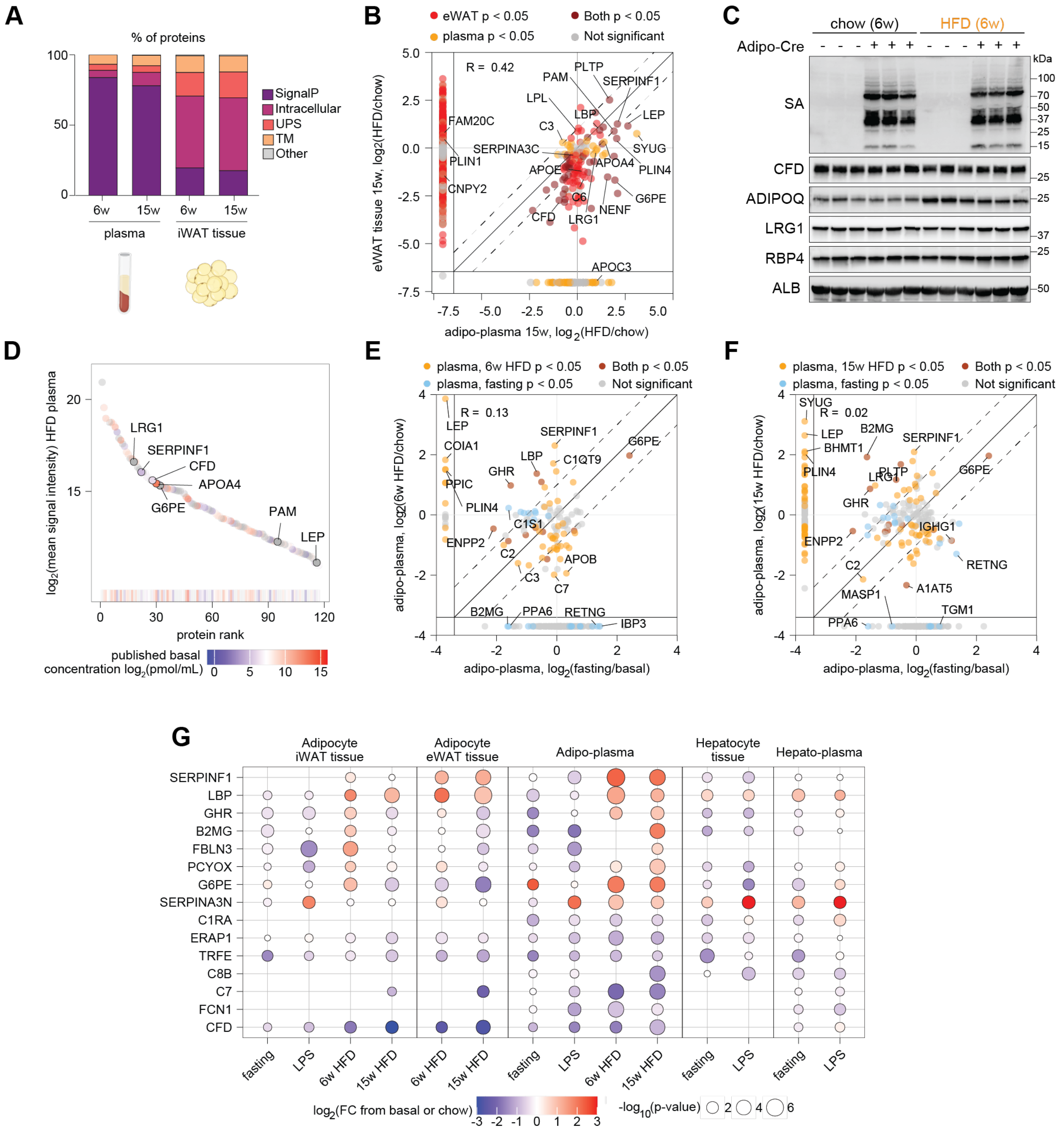

Figure S6. Characterization of adipo-plasma proteome during obesity, related to Figure 6.

(**A**) Bar plot of proteins quantified in Adipo-TurboID^{KDEL} iWAT and plasma proteomes at 6 and 15 weeks of DIO, with protein class labeled based on SignalP and OutCyte predictions.

(**B**) Scatterplot of $\log_2$ fold changes in protein expression in eWAT vs. plasma proteomes of Adipo-TurboID^{KDEL} mice following HFD feeding for 15 weeks compared to chow. Proteins quantified for only one of the conditions are shown on margins.

bioRxiv preprint doi: https://doi.org/10.64898/2026.01.11.698831; this version posted January 12, 2026. The copyright holder for this preprint (which was not certified by peer review) is the author/funder, who has granted bioRxiv a license to display the preprint in perpetuity. It is made available under aCC-BY 4.0 International license.

(**C**) Western blot validation of protein biotinylation using streptavidin-HRP and levels of known adipokines including CFD, ADIPOQ, LRG1, and RBP4 in bulk plasma samples from Adipo-TurboID^{KDEL} mice after 6 weeks of HFD feeding. Albumin (ALB) was used as a loading control.

(**D**) Rank plot of mean signal intensity values of proteins quantified by TurboID-TMT in Adipo-TurboID^{KDEL} plasma proteome following HFD feeding for 6 weeks colored by known basal plasma concentrations.

(**E-F**) Scatterplots of $\log_2$ fold changes in protein expression in plasma proteomes of Adipo-TurboID^{KDEL} mice following 48-hour fasting and HFD feeding for 6 weeks (E) or 15 weeks (F) compared to basal or chow. Proteins quantified for only one of the conditions are shown on margins.

(**G**) Dot plot of $\log_2$ fold changes and p-values of selected proteins detected in adipocytes, hepatocytes, and plasma across negative and positive energy balance conditions. Data are shown relative to own control group (e.g. fasting vs. basal, LPS vs. basal, HFD vs. chow).

bioRxiv preprint doi: https://doi.org/10.64898/2026.01.11.698831; this version posted January 12, 2026. The copyright holder for this preprint (which was not certified by peer review) is the author/funder, who has granted bioRxiv a license to display the preprint in perpetuity. It is made available under aCC-BY 4.0 International license.

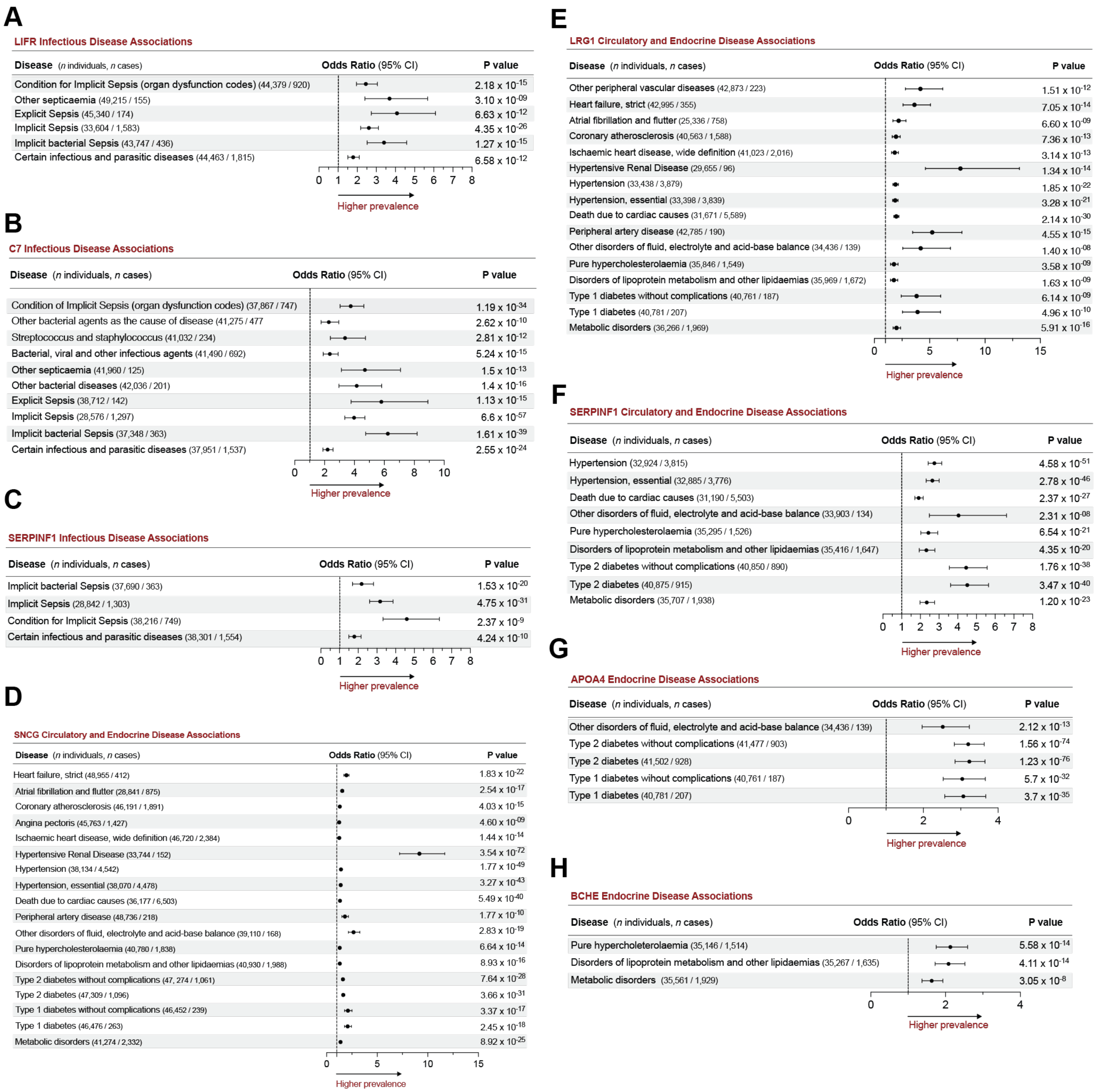

Figure S7. Odd ratios for circulatory, endocrine, and infectious disease and clinical outcomes linked with individual circulating proteins derived from adipocytes or hepatocytes in various energy balance states, related to Figure 7.

(**A-H**) Odds ratios for infectious diseases, endocrine, and/or circulatory prevalence-based outcomes linked with basal circulating levels of leukemia inhibitory factor receptor (LIFR), complement component 7 (C7), serpin family F member 1 (SERPINF1) protein, gamma synuclein (SNCG), leucine-rich glycoprotein 1 (LRG1), apolipoprotein A-IV (APOA4), and cholinesterase (BCHE) in Health and Disease Atlas based on UKBB cohort of 53,026 individuals.

bioRxiv preprint doi: https://doi.org/10.64898/2026.01.11.698831; this version posted January 12, 2026. The copyright holder for this preprint (which was not certified by peer review) is the author/funder, who has granted bioRxiv a license to display the preprint in perpetuity. It is made available under aCC-BY 4.0 International license.

(B) Supplementary Data Set Legends

Table S1: Experimental metadata and protein filtering metrics.

Details of mouse biotin labeling experiments, mass spectrometry settings, and protein quantification statistics. An ROC filter was applied for tissue experiments, while a 15% enrichment filter was applied for plasma experiments. B-cell, hepatocyte, and adipocyte enriched proteins were determined by retrieving genes from the human protein atlas in categories group enriched, cell type enriched, and cell type enhanced.

Table S2: Mean protein signal intensity across all experiments.

Signal intensity data is mean of technical replicates. Median normalization was performed for cre+ channels for each condition. Cre- channels are unnormalized values from IP2 search. Published plasma concentrations are calculated from Michaud et al 2018.

Table S3: Differential expression of proteins in the mouse secretome.

TurboID-TMT data showing protein expression changes in the mouse secretome. For every protein the $\log_2$ fold-change of the ratio between condtions was calculated. p-values were calculated with T-test for the means of two independent samples. Infinite $\log_2$(fold-change) values were replaced with the greatest non-infinite value in each direction (10.197 and -8.627)

Table S4: Proteome-phenome atlas assocations.

Protein disease associations for proteins quantified in the mouse secretome. Associations were retrieved from the proteome phenome atlas. Associations are filtered to prevalent associations with Bonferroni-corrected p-value < 0.05, in chapters I, IV, and IX.

bioRxiv preprint doi: https://doi.org/10.64898/2026.01.11.698831; this version posted January 12, 2026. The copyright holder for this preprint (which was not certified by peer review) is the author/funder, who has granted bioRxiv a license to display the preprint in perpetuity. It is made available under aCC-BY 4.0 International license.

(C) Experimental model and subject details

Mice

Experiments involving mice were approved by The Rockefeller University's Institutional Animal Care and Use Committees (Protocol numbers 21037, 19087, and 24019-H), and Yale University's Institutional Animal Care and Use Committee (Protocol number 2022-20445).

Mice were housed 2-5 per cage in a 12-hour light/12-hour dark cycle with *ad libitum* access to water and regular chow except in fasting studies where chow was restricted. We used WT male C57BL/6J mice (Jackson Laboratory 000664), Rosa26-Cas9 knockin (*Gt(ROSA)26Sor*$^{tm1.1(CAG\text{-}cas9*,\text{-}EGFP)Fezh}$/J, Jackson Laboratory 024858) Albumin-Cre (B6.Cg-Speer6-ps1$^{\mathrm{Tg(Alb\text{-}cre)21Mgn}}$/J, Jackson Laboratory 003574). CD19-Cre (B6.129P2(C)-Cd19tm$^{\mathrm{1(cre)Cgn}}$/J, Jackson Laboratory 006785), Adiponectin-Cre (B6.FVB-Tg(Adipoq-cre)1Evdr/J, Jackson Laboratory 028020), and LSL-TurboID-KDEL, which was generated in the transgenic core facility at Rockefeller University. All mouse lines are on a wild type (C57BL/6J) background. Littermates of the same sex (all male) were randomly assigned to either experimental or control groups.

Construct generation and genotyping

LSL-V5-TurboID-KDEL-IRES-eGFP was cloned by Gibson Assembly into the CTV plasmid (Addgene #15912) at the Asc1 restriction site for recombination into the Rosa26 locus. Transgenic knock-in chimeric mice were generated by ES cell injections into mouse blastocysts, at Rockefeller's Transgenic and Reproductive Technology Center. Mice were backcrossed eight generations in the C57BL/6J background before additional crosses. Mice were then bred to homozygosity without any obvious developmental defects. Primers for genotyping both homozygous and heterozygous mice were:

TurboID^{KDEL} Fwd 5' CCC GAG CCT ATC CCG CTG CTG AAC 3'

TurboID^{KDEL} Rev 5' CCC CAA TGA TCA GCT TCA CGG GTC 3'

Rosa26 Fwd 5' AAA GTC GCT CTG AGT TGT TAT 3'

Rosa26 Rev 5' GGA GCG GGA GAA ATG GAT ATG 3'

Biotin water

Biotin-containing water for basal profiling and negative energy balance experiments was prepared by dissolving solid biotin in sterile drinking water at a concentration of 0.25 mg/mL. To solubilize a higher concentration of biotin for HFD experiments (1.5 mg/mL), biotin was first dissolved in a small volume of Trizma base (3 g biotin, 20 mL, 1 M Trizma base) and then diluted in sterile drinking water to 2 L final

bioRxiv preprint doi: https://doi.org/10.64898/2026.01.11.698831; this version posted January 12, 2026. The copyright holder for this preprint (which was not certified by peer review) is the author/funder, who has granted bioRxiv a license to display the preprint in perpetuity. It is made available under aCC-BY 4.0 International license.

volume with pH returned to pH 7.5 by addition of hydrochloric acid. Mice were provided *ad libitum* access to biotin-containing water during treatment.

LPS treatment

Heterozygous (TurboID^{KDEL}/C57BL/6J) mice, with and without expression of Adiponectin- or Albumin-Cre, were used for LPS and fasting experiments. For LPS-treated animals, a frozen aliquot of LPS (Sigma Aldrich L2880, 1 mg/mL) was diluted in sterile phosphate-buffered saline (PBS) to a final concentration of 0.1 mg/mL. This solution was injected into each animal i.p. at a concentration of 1 mg/kg while saline alone was provided as a control.

Food restriction & chow consumption measurements

For fasted animals, food was removed from the hopper for 48 hours, while other groups of animals had *ad libitum* access to chow. Food intake was measured daily.

Diet-induced obesity

Homozygous (TurboID^{KDEL}/TurboID^{KDEL}) mice crossed with Adiponectin-Cre donors[1] were used for diet-induced obesity studies. All mice were group-housed (3-4 per cage), maintained at RT in 12-hour light:dark cycle (0700-1900) and allowed *ad libitum* access to water and food (chow or 60 kcal% high-fat diet, see STAR Methods Table). For early-stage obesity, male mice of both genotypes (Adipo-TurboID^{KDEL} Cre+ and Cre-) were weighed, randomly allocated to diet groups (minimum n=4 per condition) and terminated after 6 weeks of HFD feeding. An independent age-matched cohort fed HFD/chow for 6 weeks was used for glucose tolerance tests and body composition scans. For late-stage obesity, male mice were fed HFD/chow for 15 weeks starting at 7 weeks of age. Biotin was administered in drinking water for 7 days prior to plasma and tissue harvests. Biotin intake was recorded daily, and body weights were recorded weekly.

Metabolic phenotyping in diet-induced obese mice

For fasting glucose measurements and glucose tolerance tests (GTT) mice were housed individually and fasted for 6 hours in a noise-free procedure room. Circulating levels of glucose were determined from tail-derived blood using a standard glucometer. Following fasting glucose at time 0, mice were injected i.p. with bolus glucose at 1.5 g/kg in 30-second intervals and glucose measurements were taken at 15, 30, 60, 90, and 120 min post-injection. Two measurements with two separate glucometers were taken per mouse and per timepoint to ensure measurement accuracy (means are reported). Body composition (fat mass, lean mass) was assessed using rodent echoMRI.

bioRxiv preprint doi: https://doi.org/10.64898/2026.01.11.698831; this version posted January 12, 2026. The copyright holder for this preprint (which was not certified by peer review) is the author/funder, who has granted bioRxiv a license to display the preprint in perpetuity. It is made available under aCC-BY 4.0 International license.

Blood glucose measurements

A small incision was made on the mouse's tail to allow collection of blood, and blood glucose levels were measured using a Contour NextEZ glucometer.

Sample collection

Plasma sample collection: Mice were anesthetized with an overdose of isoflurane and blood was collected via cardiac puncture, using EDTA coated microcentrifuge tubes (Sarstedt), and placed on ice. Tubes were centrifuged at 1,000 *g* for 20-30 min at 4°C, followed by collection of the plasma fraction and storage at -80°C until further analysis.

Tissue sample collection: Following blood collection, mice were perfused with PBS to minimize any non-specific residual biotinylation signal, organs were then dissected out, rinsed with ice-cold PBS briefly, snap frozen in liquid nitrogen, and stored at -80°C before use.

Fixed tissues for validation in sections: Mice were anesthetized with isoflurane and an intracardiac perfusion and fixation was performed with 1X PBS followed by cold 4% PFA. All harvested samples were post-fixed in 4% PFA at 4°C for 24 h. Fixed tissue samples were washed with PBS for three times before subsequent steps.

(D) Experimental methods details

Plasma processing

For mass spectrometry, 200-400 µL of plasma was diluted to 15 mL using 1x PBS and centrifuged at 4,000 rpm for 1 h at 4°C using a 3 kDa filter (Millipore, UFC900324) twice to remove biotin, until the solution was concentrated to 0.5 mL. The final 0.5 mL solution was transferred to a new 1.5 mL tube and 0.8 mL of RIPA buffer containing protease inhibitors was added before further analysis or subsequent enrichment steps.

Tissue lysis for liver and spleen

Half of a single lobe of liver (~400 mg) and entire spleen from each animal were used for analysis. Tissues were minced using scissors and one 3 mm Tungsten carbide metal bead was added to the sample in a 2.0 mL microcentrifuge tube, containing 1 mL of cold RIPA buffer (10 mM Tris, 150 mM NaCl, 1% Triton X-100, 1% sodium deoxycholate, 0.1% SDS, pH 7.5) containing cOmplete protease inhibitor cocktail (Roche, 04693116001) and 1 mM PMSF. Tissues were then lysed using a Qiagen TissueLyser II (20 frequency/sec

bioRxiv preprint doi: https://doi.org/10.64898/2026.01.11.698831; this version posted January 12, 2026. The copyright holder for this preprint (which was not certified by peer review) is the author/funder, who has granted bioRxiv a license to display the preprint in perpetuity. It is made available under aCC-BY 4.0 International license.

for 20 min). Samples were incubated in RIPA at 4°C with end-over-end rotation for 20-30 min to boost protein extraction yield, followed by centrifugation at 14,000 rpm for 10 min, and collection of the supernatant. Protein concentration was measured using standard DC or BCA assay and subjected to further analysis or enrichment steps described below.

Protein extraction from adipose tissues

Adipose tissues (iWAT, eWAT) were harvested from anaesthetized, PBS-perfused mice and snap frozen in liquid nitrogen, then pulverized to a fine powder (Cellcrusher). Soluble proteins were extracted using NP-40-based RIPA buffer (1% NP-40, 150 mM NaCl, 0.5% deoxycholate, 0.1% SDS, 50 mM Tris-HCl pH 7.5) containing cOmplete protease inhibitor cocktail (Roche, 04693116001), and phosphatase inhibitor (Roche, 4906845001) tablets (1 tablet per 10 mL). Lysates were incubated with end-over-end rotation for 30 min at 4°C, followed by centrifugation at 8,000 rpm for 20 min at 4°C, and lipid-free soluble infranatant fractions were extracted for downstream analyses.

Immunoblotting

Protocol 1 (Figures 1, S1, 4-7)

Protein concentrations were determined using Pierce BCA assay and normalized to 3 µg/µL (iWAT, eWAT) or 9 µg/µL (plasma) in XT sample buffer, boiled at 90°C for 5 min and resolved at 30 µg/well (tissue extracts) or 90 µg/well (plasma) in 12-well 4-12% Criterion XT Bis-Tris gels in MES electrophoresis buffer. Proteins were transferred onto 0.45 µm (tissues) or 0.22 µm (plasma) ImmobilonP PVDF membranes in Tris-Glycine-MeOH buffer at 30V (4°C, overnight), blocked in biotin-free SuperBlock for 1 h at RT, and incubated with Streptavidin-HRP (SA), or primary antibodies against V5 tag, CFD, LRG1, RBP4, FABP4, ADIPOQ, ALB, and VINC, as detailed in the Key Resources Table. HRP-conjugated secondary antibodies were used for detection with enhanced chemiluminescence in ChemiDoc Imager. Tissue panel blots for V5 were run using multiple organs at the same concentration per well (30 µg total) to allow for side-by-side comparison. Vinculin (tissues), Albumin (plasma) or Coomassie Blue were used as loading controls.

Protocol 2 (Figures 2 and 3)

Protein samples were combined with SDS protein loading buffer, boiled for 5 min, and resolved by SDS-PAGE. Proteins were transferred onto a nitrocellulose membranes (0.2 µm, Bio-Rad, 1620112), which were then blocked with Odyssey blocking buffer (LICOR) or 3% w/v BSA in TBST (0.1% Tween-20 in Tris-buffered saline) for 30 min. Membranes were incubated overnight at 4°C with primary antibodies diluted in 3% (w/v) BSA in TBST, followed by four 5-min washes in TBST. Following primary antibody

bioRxiv preprint doi: https://doi.org/10.64898/2026.01.11.698831; this version posted January 12, 2026. The copyright holder for this preprint (which was not certified by peer review) is the author/funder, who has granted bioRxiv a license to display the preprint in perpetuity. It is made available under aCC-BY 4.0 International license.

incubation, membranes were probed with IRDye 800CW or IRDye 680RD (LICOR Biosciences) (1:10000 dilution) or HRP-conjugated secondary antibodies at RT for 1 h, then imaged using Bio-Rad ChemiDoc. Blots were quantified using Bio-Rad Image Lab software. HRP-labelled blots were developed using a chemiluminescence substrate (Clarity Western ECL Substrate, Bio-Rad, 1705061). Alternatively, samples were imaged using the same channels on a Bio-Rad ChemiDoc.

For Western blot validation (Figures S2I, S2J, 3G), proteins were resolved on 4-20% Mini-PROTEAN TGX stain-free gel (Bio-Rad, 4568095). Western blot band intensity was normalized to the total intensity of the corresponding lane in a stain-free gel image.

Immunofluorescence

Adipose, liver or spleen tissue was collected from anesthetized mice perfused with PBS and 4% paraformaldehyde. Liver and spleen were sunk with 30% sucrose in PBS overnight. Adipose tissue was delipidated as previously described.[2] Samples were embedded in OCT and cut into 30 µm sections at -20°C. Sample sections were blocked in 3% BSA, 1% donkey serum containing PBST (0.1% Triton X-100, 0.05% Tween-20), followed by incubation in primary antibody dilutions for 2 h at RT. After incubation with primary antibodies, samples were washed in PBST four times and then incubated in secondary antibody dilutions for 4 h at RT in blocking buffer. Samples were then washed in PBST four times and mounted with DAPI Fluoromount (Southern Biotech).

Confocal microscopy

Representative sample regions of iWAT were imaged using an inverted Zeiss LSM 980 laser scanning confocal microscope with a 40X objective lens. Liver and spleen were imaged using an inverted Leica TCS SP8 X laser scanning confocal microscope with a 40X objective lens.

Biotinylated protein enrichment

To enrich biotinylated proteins, 100 µL of streptavidin (SA) magnetic beads (Pierce, 88816) per sample was washed twice with RIPA buffer (150 mM NaCl, 1% NP-40, 0.5% sodium deoxycholate, 0.1% SDS, 50 mM Tris pH 7.5) with protease and phosphatase inhibitors. The washed slurry was then incubated with cell lysates containing the same amount of protein input (3-5 mg of protein) for all samples of that same tissue type and incubated with end-over-end rotation at 4°C overnight. Using a magnetic rack, the beads were subsequently washed twice with 1 mL of RIPA lysis buffer, once with 1 mL of 1 M KCl, once with 1 mL of 0.1 M Na_2CO_3, once with 1 mL of 2 M urea (freshly prepared) in 10 mM Tris-HCl (pH 8.0), and twice with 1 mL RIPA lysis buffer. A small volume of protein-bound beads (5% of total volume) was

bioRxiv preprint doi: https://doi.org/10.64898/2026.01.11.698831; this version posted January 12, 2026. The copyright holder for this preprint (which was not certified by peer review) is the author/funder, who has granted bioRxiv a license to display the preprint in perpetuity. It is made available under aCC-BY 4.0 International license.

removed at this point for validation by Western blot and bead-bound proteins were eluted by boiling at 95°C in 2X SDS loading buffer containing 20 mM DTT and 2 mM biotin for 15 min.

Elution of peptides from beads

To prepare proteomic samples for mass spectrometry analysis, biotinylated proteins bound to SA beads were washed with 1 mL of 50 mM Tris-HCl (pH 7.5), followed by two washes with 1 mL 2 M urea in 50 mM Tris (pH 7.5) buffer. The buffer was removed, the beads were re-suspended in 80 µL of 2 M urea in 50 mM Tris-HCl containing 1 mM DTT, 0.5 mM $CaCl_2$, and 0.8 µg trypsin (Promega, V5111), followed by incubation at 25°C for 1.5 h with shaking at 1,000 rpm. After 1.5 h, samples were placed on a magnet, and the supernatant was transferred to fresh low-binding Eppendorf tubes. Streptavidin beads were then washed twice with 60 µL of 2 M urea in 50 mM Tris (pH 7.5), and the washes were combined with the initial supernatant for subsequent reduction, alkylation, and digestion steps.

Reduction, alkylation, trypsin digestion, and desalting of peptides

Samples (200 µL total) were reduced with DTT (final concentration: 8 mM; 65°C, 20 min) and alkylated with iodoacetamide (final concentration: 20 mM; 37°C, 30 min in the dark with shaking at 1,000 rpm). An additional 0.5 µg of trypsin was added to each sample and protein digestion was carried out overnight at 25°C with shaking at 700 rpm. After overnight digestion, the sample was acidified to pH 3 by adding formic acid (FA) to a final 1% (vol/vol) FA. Samples were desalted using C18 StageTips (Pierce, 87784). C18 StageTips were first conditioned with 100 µL of 100% methanol, followed by 100 µL of solvent 1 (50:50 acetonitrile (ACN) : H_2O, 0.1% FA), and twice with 200 µL of buffer A (95% H_2O, 5% ACN, 0.1% FA). Acidified peptides were loaded onto the conditioned StageTips and washed twice with 200 µL of buffer A (95% H_2O, 5% ACN, 0.1% FA). Samples were eluted from the StageTips with 2 x 200 µL of buffer B (80% ACN/H_2O, 0.1% FA), and the solvent was removed until completely dry using SpeedVac vacuum concentrator.

TMT labeling for multiplexed quantitative proteomics

Peptides were resuspended in 100 µL of 30% ACN in 200 mM EPPS (pH 8.0). Samples were vortexed, spun down, and sonicated in a water bath for 5 min. After brief centrifugation, 3 µL of the corresponding TMTpro 16-plex reagent (Thermo Fisher, A44521) was added to each tube (20 µg/µL in dry ACN). The samples were vortexed, spun down, and left at room temperature for 1 h. To quench the labeling, 3 µL of 5% hydroxylamine was added to each sample. Samples were vortexed, spun down, and left at room temperature for 15 min. Samples were then acidified with 5 µL of FA, combined into two low binding 1.5 mL Eppendorf tubes, and dried using SpeedVac vacuum concentrator, leaving gel-like salt residues.

bioRxiv preprint doi: https://doi.org/10.64898/2026.01.11.698831; this version posted January 12, 2026. The copyright holder for this preprint (which was not certified by peer review) is the author/funder, who has granted bioRxiv a license to display the preprint in perpetuity. It is made available under aCC-BY 4.0 International license.

Desalting of pooled multiplexed samples

Following TMT labeling, samples were resuspended in 500 µL of buffer A (95% H_2O, 5% ACN, 0.1% FA). An additional 20 µL of 20% FA was added to ensure the sample remained acidic. The Sep-Pak C18 cartridge was conditioned by adding 1 mL 100% ACN three times and equilibrated using 1 mL buffer A (95% H_2O, 5% ACN, 0.1% FA) three times. Samples were added slowly at a rate of 1 drop/sec, and the flow through was collected and re-loaded a second time. To desalt the samples, 1 mL of buffer A (95% H_2O, 5% ACN, 0.1% FA) was added to the cartridge three times. Samples were eluted by adding 1 mL of buffer B (80% ACN, 20% H_2O, 0.1% FA). The eluate was then dried using SpeedVac vacuum concentrator.

Pre-fractionation by HPLC

Dried desalted samples were resuspended in 500 µL buffer A (95% H_2O, 5% ACN, 0.1% FA) and loaded on a high-pressure liquid chromatography (HPLC) for offline pre-fractionation. Samples were separated on a C18 column by reverse phase HPLC, starting in a high pH buffer containing 10 mM ammonium bicarbonate over an increasing acetonitrile gradient (0-100%). Samples were collected into a deep 96-well plate, pooled across elution times (12 fractions for tissue, 4 fractions for plasma), and then dried using SpeedVac vacuum concentrator. Because the adipo-plasma and hepato-plasma proteomes were expected to be lower in abundance than the intracellular ER-associated proteomes, we pooled HPLC-fractionated samples into four final fractions to optimize coverage and enhance detection of low abundance factors.

Liquid chromatography tandem mass spectrometry analysis (LC-MS/MS/MS)

Samples were re-suspended in 10 µL of buffer A and briefly sonicated before analysis by liquid chromatography tandem mass spectrometry (7.5 µL injection) using Orbitrap Eclipse mass spectrometer (Thermo Fisher) with UltiMate 3000 Series Rapid Separation LC system. Peptides were eluted onto a 2 µm, 100 Å, 75µm x 25 cm capillary column at 0.25 µL/min flow rate and separated using the following gradient: 5% LC-MS buffer B (ACN, 0.1% FA) in LC-MS buffer A (H_2O, 0.1% FA) from 0-10 min, 5%-20% buffer B from 20-120 min, 20%-45% buffer B from 120-140 min, 45%-95% buffer B from 140-145 min, 95% buffer B from 145-147 min, 5% buffer B from 147-149 min, 95% buffer B from 149-151 min, 5% buffer B from 151-160 min).

The following scan sequence was used: (1) MS1 master scan (Orbitrap analysis, resolution 120,000, scan range 400-1,600 m/z, RF lens 40%, normalized AGC Target 250%, automatic maximum injection time, profile mode with enabled dynamic exclusion (repeat count 1, duration 60 s); (2) Selection of top 20 ions for MS2 analysis; (3) MS2 analysis, which consisted of quadrupole isolation (isolation window 0.7 m/z) of precursor ion followed by collision-induced dissociation (CID) in the ion trap (fixed collision

bioRxiv preprint doi: https://doi.org/10.64898/2026.01.11.698831; this version posted January 12, 2026. The copyright holder for this preprint (which was not certified by peer review) is the author/funder, who has granted bioRxiv a license to display the preprint in perpetuity. It is made available under aCC-BY 4.0 International license.

energy mode, normalized collision energy 35%, maximum CID activation time 10 ms, Activation Q 0.25). Except for diet-induced obese and chow-fed iWAT and eWAT analyses, all runs used real-time search with a 2017 mouse FASTA database and included static modifications for cysteine carbamidomethylation (Δ Mass 57.0215), lysine-, and N-terminal amine modification with TMTpro 16-plex reagents (Δ Mass 304.2071), as well as variable modifications for methionine oxidation (Δ Mass 15.9949). Maximum missed cleavages were set to 2, with maximum variable modifications per peptide at 1.

RNA preparation and quantitative PCR

Total RNA was extracted from white fat tissue, spleen or liver using TRIzol (Invitrogen) along with QIAGEN RNeasy mini kits. For quantitative PCR (qPCR) analysis, RNA was reverse transcribed using the QuantiTect Reverse Transcription Kit (Qiagen). cDNA was used in qPCR reactions containing SYBR-green, fluorescent dye (ABI). Relative mRNA expression was determined by normalizing to the geometric mean of *Ppib* (cyclophilin B) and *Tbp* (TATA-box binding protein) levels using the $\Delta\Delta C_t$ method. Primer sequences are:

Cyclo/*Ppib* Fwd 5' GGA GAT GGC ACA GGA GGA A 3'

Cyclo/*Ppib* Rev 5' GCC CGT AGT GCT TCA GCT T 3'

Tbp Fwd 5' GGG TAT CTG CTG GCG GTT T 3'

Tbp Rev 5' TGA AAT AGT GAT GCT GGG CAC T 3'

Pam Fwd 5' CTG GGG TCA CAC CTA AAG AGT 3'

Pam Rev 5' ATG AGG GCA TGT TGC ATC CAA 3'

Ppic Fwd 5' TGA CGG ACA AGG TCT TCT TTG A 3'

Ppic Rev 5' CAG AGC CAC GAA GTT TTC CAC 3'

SerpinF Fwd 5' GCC CTG GTG CTA CTC CTC T 3'

SerpinF Rev 5' CGG ATC TCA GGC GGT ACA G 3'

Cdnf Fwd 5' CTT TTG CGC CGG GTT TTG TAT 3'

Cdnf Rev 5' AGG GAG TTG TAG AAT CGG TCT AA 3'

Sncg Fwd 5' AAA GAC CAA GCA GGG AGT AAC G 3'

Sncg Rev 5' GAC CAC GAT GTT TTC AGC CTC 3'

Nenf Fwd 5' GAA GGG AGT GGT GTT CGA TGT 3'

Nenf Rev 5' GTG TCG TGA GTG AGG TCT GC 3'

Flst1 Fwd 5' CAC GGC GAG GAG GAA CCT A 3'

Flst1 Rev 5' TCT TGC CAT TAC TGC CAC ACA 3'

Gpr50 Fwd 5' AGA GCA ACA TGG GAC CTA CAA 3'

Gpr50 Rev 5' GCC AGA ATT TCG GAG CTT CTT G 3'

bioRxiv preprint doi: https://doi.org/10.64898/2026.01.11.698831; this version posted January 12, 2026. The copyright holder for this preprint (which was not certified by peer review) is the author/funder, who has granted bioRxiv a license to display the preprint in perpetuity. It is made available under aCC-BY 4.0 International license.

(E) Quantification and statistical analysis

Statistical analyses

Statistical analyses were performed using GraphPad Prism and R package. Two-way ANOVA followed by Tukey's multiple comparisons test was applied to determine the statistical differences among groups across different timepoints. Non-repeated measure data were compared to basal with ordinary one-way ANOVA and Šidák multiple comparisons test. Unpaired two-tailed Student's t test was applied to determine the statistical differences for gene expression and protein levels. The results of statistical analyses for each experiment can be found in corresponding figure legends. All values are reported as mean ± SEM. P-values below 0.05 were considered significant throughout the study.

Data processing for proteomic experiments

Raw data files were processed using RAW converter (available at github.com/proteomicsyates/RawConverter) and the resulting MS1, MS2, and MS3 files were uploaded to the Integrated Proteomics Pipeline (IP2). Peptide identification was performed against a reverse concatenated, non-redundant version of the Mouse Uniprot database (release-2017_07) using the ProLuCID algorithm. Static modifications for TMT labeling were set for N-termini and lysine residues (+304.2071 Da for TMT 16-plex). Search results were filtered with DTASelect (v2.0) to achieve a peptide-level false discovery rate below 1%. MS3 reporter ions were used for quantification with a mass tolerance of 20 ppm via the IP2 platform.

Filtering and normalization of proteomic data

Experiments were processed individually with the following filters: removal of non-unique peptides, removal of half-tryptic peptides, removal of peptides with more than two internal missed cleavages, removal of peptides with low (< 5,000) average of reporter ion intensities for all pairs of channels, and peptides with high variation between all pairs of channels (coefficient of variation > 0.5). Additionally, keratin proteins and proteins whose human orthologs were quantified in over 200 experiments in the CRAPome[3] database were labelled as contaminants and excluded from analysis.

Median normalization was performed on signal intensities for Cre+ channels within each experiment. True positive proteins (defined in Receiver operating characteristic analysis) with 2+ peptides were used to calculate the median per channel and normalization factors were calculated by dividing the median of all Cre+ channel medians by each individual channel's median. Each Cre+ channel was then multiplied by its corresponding normalization factor.

bioRxiv preprint doi: https://doi.org/10.64898/2026.01.11.698831; this version posted January 12, 2026. The copyright holder for this preprint (which was not certified by peer review) is the author/funder, who has granted bioRxiv a license to display the preprint in perpetuity. It is made available under aCC-BY 4.0 International license.

Receiver operating characteristic (ROC) analysis

For tissue samples, receiver operating characteristic (ROC) curves were used to set the TMT ratio cutoffs that would maximize the retention of true positives, while minimizing the retention of false positives in each case, as described by the Ting lab.[4,5]

Proteins were labeled as true positive based on subcellular location annotations retrieved from uniprot.org. Proteins with any of the following annotations were labeled as true positive (case-insensitive): "secreted", "endoplasmic reticulum", "rough endoplasmic reticulum". Proteins were labeled as false positive if they were found in the human mitomatrix list from supplementary table S1 from Rhee et al., 2013.[6] Additionally, proteins with signal peptide annotation were removed from the false positive list. Proteins labeled as both true positive and false positive through this approach were reassigned as true positive.

Gene Ontology term enrichment analysis

Gene ontology enrichment analysis was performed on the significant upregulated or downregulated proteins (fold-change > 1.5 and p-value < 0.05) using Fisher's exact test implemented in GOATOOLS against the Gene Ontology (goslim_generic.obo, downloaded on April 24, 2024). An experiment-specific background (i.e. all proteins quantified in the experiment) was used for all analyses except for the cross-experiment analysis (Figure S2F) where the full mouse proteome was used. GO Biological Process terms were mapped to GOslim terms using the GOATOOLS mapslim.py script. After analysis, terms with a Benjamini-Hochberg corrected p-value less than 0.05 were retained.

Absolute plasma concentrations

Concentration of plasma proteins quantified by multiple reaction monitoring mass spectrometry in Michaud et *al*. (2018)[7] were compared with proteins detected in the present study. Of the 385 target analytes detected in C56BL/6J or C57BL/6BR mice in supplementary tables 4, 6, and 7 of Michaud *et al*. (2018), 243 were detected in one or more of the basal, negative energy balance, and obesity conditions. Values were displayed for comparison in Figures 3, S3, 6, and S6, and are available in Tables S2 and S3.

UK Biobank

UK Biobank protein-disease associations for prevalent diseases were retrieved from the protein phenome atlas[8] (proteome-phenome-atlas.com). P-values for diseases were corrected through the Bonferroni correction and only significant associations (Bonferroni adjusted p < 0.05) are shown. Proteins with significant disease associations in UKBB chapters I, IV or IX orthologous to TurboID plasma proteins significantly regulated by low- or high-energy balance are listed in Table S4.

bioRxiv preprint doi: https://doi.org/10.64898/2026.01.11.698831; this version posted January 12, 2026. The copyright holder for this preprint (which was not certified by peer review) is the author/funder, who has granted bioRxiv a license to display the preprint in perpetuity. It is made available under aCC-BY 4.0 International license.

[F] KEY RESOURCES TABLE

REAGENT or RESOURCE	SOURCE	IDENTIFIER / RRID	
**Antibodies**			
Streptavidin, HRP-linked	Cell Signaling	Cat. 3999S	AB_10830897
anti-V5 tag	Abcam	Cat. ab206562	AB_3719924
anti-Human IgG, HRP-linked	Abcam	Cat. ab6858	AB_955433
anti-mAcrp30 (adiponectin)	R&D Systems	Cat. AF1119	AB_2221770
anti-Goat IgG, HRP-linked	Thermo Fisher	Cat. A15999	AB_2534673
Streptavidin, Alexa 647	BioLegend	Cat. 405237	
anti-mAlbumin	Cell Signaling	Cat. 4929S	AB_2225785
anti-Rabbit IgG, HRP-linked	Cell Signaling	Cat. 7074S	AB_2099233
anti-BTK	Cell Signaling	Cat. 8547S	AB_10950506
anti-Ces1d	Santa Cruz Biotechnology	Cat. sc-374160	AB_10988772
IRDye 800CW Goat anti-Mouse IgG Secondary	LI-COR	Cat. 926-32210	AB_621842
anti-Complement C6	Invitrogen	Cat. PA5-117369	AB_2901999
IRDye 680CW Goat anti-Rabbit IgG Secondary	LI-COR	Cat. 926-68071	AB_10956166
anti-SerpinA3N	R&D Systems	Cat. AF4709	AB_2270116
IRDye 680RD Donkey anti-Goat IgG Secondary	LI-COR	Cat. 926-68074	AB_10956736
IRDye 800CW Streptavidin	LI-COR	Cat. 926-32230	
anti-mFactor D (adipsin)	R&D Systems	Cat. AF5430	AB_1655868
anti-LRG1	Sigma-Aldrich	Cat. HPA001888	AB_1079276
anti-FABP4	R&D Systems	Cat. AF1443-SP	
anti-mRetinol binding protein 4	R&D Systems	Cat. AF3476	AB_2167682
anti-Sheep IgG, HRP-linked	Thermo Fisher	Cat. A16041	AB_2534715
anti-Vinculin	Cell Signaling	Cat. 4650S	AB_10559207
**Chemicals, peptides, and recombinant proteins**			
Biotin	Sigma-Aldrich	Cat. B4501	
NP-40	Millipore	Cat. 492016	
cOmplete Protease Inhibitor Cocktail tablets	Roche	Cat. 04693159001	

bioRxiv preprint doi: https://doi.org/10.64898/2026.01.11.698831; this version posted January 12, 2026. The copyright holder for this preprint (which was not certified by peer review) is the author/funder, who has granted bioRxiv a license to display the preprint in perpetuity. It is made available under aCC-BY 4.0 International license.

REAGENT or RESOURCE	SOURCE	IDENTIFIER / RRID
PhosphoSTOP	Roche	Cat. 04906837001
XT MES Buffer	Bio-Rad	Cat. 1610789
Criterion 4-12% Bis-Tris 12-well gels	Bio-Rad	Cat. 3450123
Tris/Glycine transfer Buffer	Bio-Rad	Cat. 1610771
SuperBlock T20 blocking buffer	Thermo Scientific	Cat. 37536
0.45 µm PVDF membrane	Millipore	Cat. IPVH00010
Enhanced Chemiluminescence (ECL)	PerkinElmer	Cat. NEL104001EA
Paraformaldehyde	Sigma-Aldrich	Cat. P6148
Dichloromethane	Fisher Scientific	Cat. AA39116K2
Hydrogen peroxide, 30%	Fisher Scientific	Cat. H325100
Heparin	Sigma-Aldrich	Cat. H3393
Dibenzyl ether	Sigma-Aldrich	Cat. 108014
Pierce Streptavidin Magnetic Beads	Thermo Scientific	Cat. 88817
Urea	Supelco	Cat. 108487
Iodoacetamide	Sigma-Aldrich	Cat. I1149
Dithiothreitol (DTT)	Fisher Scientific	Cat. BP172-25
Trypsin, sequencing grade	Promega	Cat. V5111
Acetonitrile, LC/MS grade	Fisher Scientific	Cat. A955
Hydroxylamine solution	Sigma-Aldrich	Cat. 467804
EPPS	Alfa Aesar	Cat. A13714-22
TMTpro 16-plex Label Reagent Set	Thermo Scientific	Cat. A44521
Lipopolysaccharide (LPS)	Sigma-Aldrich	Cat. L2880
Nitrocellulose membrane, 0.2 µm	Bio-Rad	Cat. 1620112
Intercept (TBS) Blocking Buffer	LI-COR	Cat. 927-60001
D-(+)-Glucose	Sigma-Aldrich	Cat. G8270
**Critical commercial assays**		
DC assay kit	Bio-Rad	Cat. 5000111
BCA assay kit	Thermo Scientific	Cat. 23228
Pierce C18 pipette tips	Thermo Scientific	Cat. 87784
Sep-Pak Vac C18 cartridges	Waters	Cat. WAT054955

bioRxiv preprint doi: https://doi.org/10.64898/2026.01.11.698831; this version posted January 12, 2026. The copyright holder for this preprint (which was not certified by peer review) is the author/funder, who has granted bioRxiv a license to display the preprint in perpetuity. It is made available under aCC-BY 4.0 International license.

REAGENT or RESOURCE	SOURCE	IDENTIFIER / RRID
Glucose Meter, Glucose Strips	Nova Max Plus Blood Glucose Monitoring System	Cat. 8548043524
QuantiTect Reverse Transcription Kit	QIAGEN	Cat. 205311
SYBR GreenER qPCR SuperMix	Invitrogen	Cat. 11760500
**Deposited data**		
All raw proteomic data has been uploaded to PRIDE	This paper	PRIDE: TBD
**Experimental Models: Organisms/strains**		
TurboID^{KDEL}	This paper	
Albumin-Cre (B6.Cg-Speer6-ps1$^{Tg(Alb-cre)21Mgn}$/J)	The Jackson Laboratory	Strain 003574
Adiponectin-Cre (B6.FVB-Tg(Adipoq-cre) 1Evdr/J)	The Jackson Laboratory	Strain 028020
CD19-Cre (B6.129P2(C)-Cd19$^{tm1(cre)Cgn}$/J)	The Jackson Laboratory	Strain 006785
Wild-type (C57BL/6J)	The Jackson Laboratory	Strain 000664
**Recombinant DNA**		
CTV plasmid	Addgene	Cat. 15912
**Software and algorithms**		
IP2 and ProLuCID	Integrated Proteomics Applications	Xu et al., *J Proteomics* (2015)[9]
RawConverter, version 1.2.0.1	Yates Lab, Scripps Research	He et al., *Anal Chem* (2015)[10]
NumPy (v1.22.4)	Van der Walt et al., 2010[11]	numpy.org
Pandas (v1.4.3)	McKinney, 2010[12]	pandas.pydata.org
ggplot2 (v3.3.5)	Wikham, 2016[13]	ggplot2.tidyverse.org
ComplexHeatmap (v2.10.0)	Gu, 2022[14]	github.com/jokergoo/ComplexHeatmap
GOATOOLS (v1.3.1)	Klopfenstein et al. 2018[15]	github.com/tanghaibao/goatools

bioRxiv preprint doi: https://doi.org/10.64898/2026.01.11.698831; this version posted January 12, 2026. The copyright holder for this preprint (which was not certified by peer review) is the author/funder, who has granted bioRxiv a license to display the preprint in perpetuity. It is made available under aCC-BY 4.0 International license.

REAGENT or RESOURCE	SOURCE	IDENTIFIER / RRID
scikit-learn (v1.0.2)	Pedregosa et al., 2011[16]	scikit-learn.org/stable
ggrepel (v0.9.1)	Slowikowski, 2024	github.com/slowkow/ggrepel
**Other**		
Rodent regular chow diet	PicoLab	Cat. 5053
Rodent diet with 60 kcal% Fat	Research Diets	Cat. D12492
Rodent water bottle nozzle, 30mm, short straight	Vision Racks & Cages	Type E
Microvette EDTA capillary blood collection tubes	Sarstedt	Cat. 16.444.100
SignalP (v6.0)	Emanuelsson, 2007[17]	services.healthtech.dtu.dk/services/SignalP-6.0/

(G) REFERENCES

(1) Eguchi, J.; Wang, X.; Yu, S.; Kershaw, E. E.; Chiu, P. C.; Dushay, J.; Estall, J. L.; Klein, U.; Maratos-Flier, E.; Rosen, E. D. Transcriptional Control of Adipose Lipid Handling by IRF4. *Cell Metab.* **2011**, *13* (3), 249–259. https://doi.org/10.1016/j.cmet.2011.02.005.

(2) Lin, Z.; Chi, J.; Cohen, P. A Clearing Method for Three-Dimensional Imaging of Adipose Tissue. In *Brown Adipose Tissue*; Guertin, D. A., Wolfrum, C., Eds.; Methods in Molecular Biology; Springer US: New York, NY, 2022; Vol. 2448, pp 73–82. https://doi.org/10.1007/978-1-0716-2087-8_4.

(3) Mellacheruvu, D.; Wright, Z.; Couzens, A. L.; Lambert, J.-P.; St-Denis, N. A.; Li, T.; Miteva, Y. V.; Hauri, S.; Sardiu, M. E.; Low, T. Y.; Halim, V. A.; Bagshaw, R. D.; Hubner, N. C.; Al-Hakim, A.; Bouchard, A.; Faubert, D.; Fermin, D.; Dunham, W. H.; Goudreault, M.; Lin, Z.-Y.; Badillo, B. G.; Pawson, T.; Durocher, D.; Coulombe, B.; Aebersold, R.; Superti-Furga, G.; Colinge, J.; Heck, A. J. R.; Choi, H.; Gstaiger, M.; Mohammed, S.; Cristea, I. M.; Bennett, K. L.; Washburn, M. P.; Raught, B.; Ewing, R. M.; Gingras, A.-C.; Nesvizhskii, A. I. The CRAPome: A Contaminant Repository for Affinity Purification-Mass Spectrometry Data. *Nat. Methods* **2013**, *10* (8), 730–736. https://doi.org/10.1038/nmeth.2557.

(4) Branon, T. C.; Bosch, J. A.; Sanchez, A. D.; Udeshi, N. D.; Svinkina, T.; Carr, S. A.; Feldman, J. L.; Perrimon, N.; Ting, A. Y. Efficient Proximity Labeling in Living Cells and Organisms with TurboID. *Nat. Biotechnol.* **2018**, *36* (9), 880–887. https://doi.org/10.1038/nbt.4201.

(5) Cho, K. F.; Branon, T. C.; Udeshi, N. D.; Myers, S. A.; Carr, S. A.; Ting, A. Y. Proximity Labeling in Mammalian Cells with TurboID and Split-TurboID. *Nat. Protoc.* **2020**, *15* (12), 3971–3999. https://doi.org/10.1038/s41596-020-0399-0.

(6) Rhee, H.-W.; Zou, P.; Udeshi, N. D.; Martell, J. D.; Mootha, V. K.; Carr, S. A.; Ting, A. Y. Proteomic Mapping of Mitochondria in Living Cells via Spatially Restricted Enzymatic Tagging. *Science* **2013**, *339* (6125), 1328–1331. https://doi.org/10.1126/science.1230593.

(7) Michaud, S. A.; Sinclair, N. J.; Pětrošová, H.; Palmer, A. L.; Pistawka, A. J.; Zhang, S.; Hardie, D. B.; Mohammed, Y.; Eshghi, A.; Richard, V. R.; Sickmann, A.; Borchers, C. H. Molecular Phenotyping of Laboratory Mouse Strains Using 500 Multiple Reaction Monitoring Mass Spectrometry Plasma Assays. *Commun. Biol.* **2018**, *1* (1), 78. https://doi.org/10.1038/s42003-018-0087-6.

bioRxiv preprint doi: https://doi.org/10.64898/2026.01.11.698831; this version posted January 12, 2026. The copyright holder for this preprint (which was not certified by peer review) is the author/funder, who has granted bioRxiv a license to display the preprint in perpetuity. It is made available under aCC-BY 4.0 International license.

(8) Deng, Y.-T.; You, J.; He, Y.; Zhang, Y.; Li, H.-Y.; Wu, X.-R.; Cheng, J.-Y.; Guo, Y.; Long, Z.-W.; Chen, Y.-L.; Li, Z.-Y.; Yang, L.; Zhang, Y.-R.; Chen, S.-D.; Ge, Y.-J.; Huang, Y.-Y.; Shi, L.-M.; Dong, Q.; Mao, Y.; Feng, J.-F.; Cheng, W.; Yu, J.-T. Atlas of the Plasma Proteome in Health and Disease in 53,026 Adults. *Cell* **2025**, *188* (1), 253-271.e7. https://doi.org/10.1016/j.cell.2024.10.045.

(9) Xu, T.; Park, S. K.; Venable, J. D.; Wohlschlegel, J. A.; Diedrich, J. K.; Cociorva, D.; Lu, B.; Liao, L.; Hewel, J.; Han, X.; Wong, C. C. L.; Fonslow, B.; Delahunty, C.; Gao, Y.; Shah, H.; Yates, J. R. ProLuCID: An Improved SEQUEST-like Algorithm with Enhanced Sensitivity and Specificity. *J. Proteomics* **2015**, *129*, 16–24. https://doi.org/10.1016/j.jprot.2015.07.001.

(10) He, L.; Diedrich, J.; Chu, Y.-Y.; Yates, J. R. Extracting Accurate Precursor Information for Tandem Mass Spectra by RawConverter. *Anal. Chem.* **2015**, *87* (22), 11361–11367. https://doi.org/10.1021/acs.analchem.5b02721.

(11) Van Der Walt, S.; Colbert, S. C.; Varoquaux, G. The NumPy Array: A Structure for Efficient Numerical Computation. **2011**. https://doi.org/10.48550/ARXIV.1102.1523.

(12) McKinney, W. Data Structures for Statistical Computing in Python; Austin, Texas, 2010; pp 56–61. https://doi.org/10.25080/Majora-92bf1922-00a.

(13) Wickham, H. Data Analysis. In *ggplot2*; Use R!; Springer International Publishing: Cham, 2016; pp 189–201. https://doi.org/10.1007/978-3-319-24277-4_9.

(14) Gu, Z.; Eils, R.; Schlesner, M. Complex Heatmaps Reveal Patterns and Correlations in Multidimensional Genomic Data. *Bioinformatics* **2016**, *32* (18), 2847–2849. https://doi.org/10.1093/bioinformatics/btw313.

(15) Klopfenstein, D. V.; Zhang, L.; Pedersen, B. S.; Ramírez, F.; Warwick Vesztrocy, A.; Naldi, A.; Mungall, C. J.; Yunes, J. M.; Botvinnik, O.; Weigel, M.; Dampier, W.; Dessimoz, C.; Flick, P.; Tang, H. GOATOOLS: A Python Library for Gene Ontology Analyses. *Sci. Rep.* **2018**, *8* (1), 10872. https://doi.org/10.1038/s41598-018-28948-z.

(16) Fabian Pedregosa, Gaël Varoquaux, Alexandre Gramfort, Vincent Michel, Bertrand Thirion, Olivier Grisel, Mathieu Blondel, Peter Prettenhofer, Ron Weiss, Vincent Dubourg, Jake Vanderplas, Alexandre Passos, David Cournapeau, Matthieu Brucher, Matthieu Perrot, and Édouard Duchesnay. 2011. Scikit-learn: Machine Learning in Python. J. Mach. Learn. Res. 12, null (2/1/2011), 2825–2830.

(17) Emanuelsson, O.; Brunak, S.; Von Heijne, G.; Nielsen, H. Locating Proteins in the Cell Using TargetP, SignalP and Related Tools. *Nat. Protoc.* **2007**, *2* (4), 953–971. https://doi.org/10.1038/nprot.2007.131.
